# Reaction to Endoplasmic Reticulum Stress *via* ATF6 in Amyotrophic Lateral Sclerosis Deteriorates With Aging

**DOI:** 10.3389/fnagi.2019.00005

**Published:** 2019-01-25

**Authors:** Tino Prell, Beatrice Stubendorff, Thanh Tu Le, Nayana Gaur, Vedrana Tadić, Annekathrin Rödiger, Otto W. Witte, Julian Grosskreutz

**Affiliations:** ^1^Hans Berger Department of Neurology, Jena University Hospital, Jena, Germany; ^2^Center for Healthy Aging, Jena University Hospital, Jena, Germany

**Keywords:** endoplasmic reticulum stress, activating transcription factor 6, unfolded protein response, peripheral blood mononuclear cells, aging, progression

## Abstract

Amyotrophic lateral sclerosis (ALS) is a multisystemic neurodegenerative disorder. Given that peripheral blood mononuclear cells (PBMCs) serve as a “window to the central nervous system” we aimed to answer whether endoplasmic reticulum (ER) stress in ALS-PBMCs is related to disease aggressiveness. We studied ER stress in the PBMCs of 49 patients with ALS and 31 age- and sex-matched healthy controls. The expression of a main ER stress marker, activating transcription factor 6 (ATF6), was significantly higher in ALS compared to controls, but did not correlate with age, disease severity, disease duration and disease progression rate. When ATF6 expression levels were plotted against relative D50 (rD50)-derived disease phases derived from the D50 ALS model, two distinct clusters of patients were observed: cluster 1, with progressively increasing ATF6 expression levels and cluster 2, which demonstrated stable ATF6 expression over the disease course. Individuals in the two clusters did not significantly differ in terms of ALS Functional Rating Scale-Revised (ALSFRS-R), disease aggressiveness, disease duration and subtype. However, patients with the increasing ATF6 level were significantly younger, indicating that aging processes might be related to ER stress in ALS. Our data suggest that the reaction to ER stress during disease course may be compromised in older patients with ALS.

## Introduction

Amyotrophic lateral sclerosis (ALS) is a fatal multisystemic neurodegenerative disorder characterized by motor neuron degeneration as well as cognitive and behavioral deficits. Identifying factors that contribute to rapid progression may prove valuable for the development of therapeutic options. Disease progression was related to deficiencies in protein degradation and axonal transport mechanisms in ALS mouse models (Nardo et al., 2013). Moreover, several studies indicate that endoplasmic reticulum (ER) stress is involved in the pathogenesis of ALS (Atkin et al., 2008; Prell et al., 2012). ER stress occurs when ER homeostasis is disturbed and misfolded proteins accumulate in the ER. To cope with this stress, cells activate the unfolded protein response (UPR). In doing so, the activating transcription factor 6 (ATF6) is cleaved in the Golgi apparatus and the resulting N-terminal fragment (p50-ATF6α) translocates to the nucleus. Here, it regulates genes related to protein quality control, protein translocation, folding and additional components of the ER associated protein degradation pathway (Xiang et al., 2017). Similar to ATF6, other UPR markers, such as X-Box-Binding Protein 1, inositol requiring enzyme-1 get activated during ER stress (Rahman et al., 2017). All of these components of the UPR work in concert to help ameliorate ER stress. However, ongoing and chronic ER stress can lead to cell death *via* apoptosis.

Given the multisystemic nature of ALS, disease processes are also reflected in the periphery. Peripheral blood mononuclear cells (PBMCs) undergo changes in immunophenotype, decreased mtDNA gene expression, increased nitrative stress and calcium dysregulation (Curti et al., 1996; Mantovani et al., 2009; Nardo et al., 2009; Ladd et al., 2014). The advantages of low invasiveness for the patient, the consequent greater availability of samples for large clinical studies and the simple laboratory procedures involved make PBMCs a promising biomarker in ALS. The aim of the study was two-fold: First, to determine whether activated UPR can be observed in PBMCs from ALS patients. Second, to evaluate if ER stress is related to disease progression.

## Materials and Methods

The study was approved by the local ethics committee of the Jena University Hospital (number: 3633-12/12) and all subjects gave written informed consent. The study was conducted according to the principles of the Helsinki Declaration of 1975, as revised in 1983. Patients with systemic infection (increased leucocytes, increased c-reactive protein) were not included. In total, 49 patients with sporadic definite, probable, laboratory-supported probable, or possible ALS (diagnosed according to the revised El Escorial criteria as having at least possible ALS), and 31 age- and sex matched healthy controls were enrolled. Disease severity was assessed with the revised ALS Functional Rating Scale (ALSFRS-R). Disease progression was modeled using the D50 model (Poesen et al., 2017). The model is based on the observation that progression of ALS is not linear; after symptom onset the ALSFRS-R does not drop immediately but decays slowly first. Then a period of uniform progression follows and with increasing disability, ALSFRS-R seems to reach a plateau again. The transition between two states, i.e., full health to maximum disease, was described as: *y* = *Amax* + (*Amin − Amax*) 1 + *e* (*x* − *D*50) *dx*. More simply, we used the known limits of the ALSFRS-R: *y* = 48 + *e* (*x* − *D*50) *dx*. We set the following constraints: *Amax* = 48 (maximum ALSFRS-R before onset); *Amin* = 0 (theoretical minimum ALSFRS-R); D50 = time point when ALSFRS-R drops to 24; *dx* = slope of ALSFRS-R decrease. Here, D50 is a summative descriptor of disease aggressiveness. The relative D50 (rD50) describes individual disease course covered in reference to D50. rD50 is an open-ended reference point where 0 signifies disease onset and 0.5 indicates the time-point of halved functionality. Using rD50 allows sampling ALS as an entity, because the different disease courses of patients are aligned in a normalized framework. It is important to notice that rD50 describes continuous disease phases and is not equal to disease duration.

PBMC samples were isolated and prepared as described before (Liu et al., 2016). After blocking with 1% bovine serum albumin, the filters were incubated overnight with the primary ATF6 antibody (Thermo Fisher PA-5, 1:500) and GAPDH (Cell Signaling, 1:1,000). Secondary antisera comprised horseradish peroxidase conjugated goat anti-rabbit IgG (Santa Cruz sc-2030, 1:2,000). Immunoreactivity was visualized using an enhanced chemiluminescence ECL detection system (BioRad, Germany). The intensity of the bands was obtained and adjusted to GAPDH expression.

All statistical analyses were performed with the SPSS software package (Version 23). Data are given as mean ± standard deviation. Independent sample *t*-tests and Mann-Whitney *U* tests were used to conduct between-group comparisons for normally and non-normally distributed data, respectively. Correlation was tested using Pearson’s correlation for normal distribution and Spearman’s correlation for non-normal distributed data. The optimal cut off level for dichotomizing values for the determination of sensitivity and specificity was selected as the situation maximizing the Youden index. The receiver operating characteristic (ROC) curve was used for a graphic visualization of the variation in the cutoff values. All values are given as mean and standard deviation. Significance level was set to *p* < 0.05.

## Results

**Figure 1:**
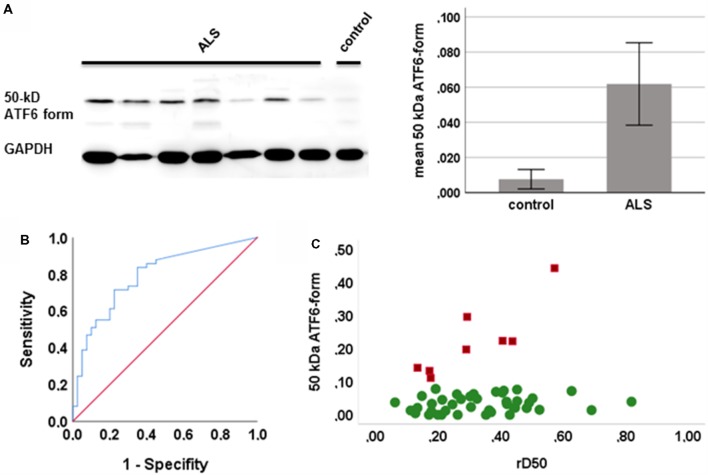
Expression of the active form of activating transcription factor 6 (ATF6) in human peripheral blood mononuclear cells (PBMCs). **(A)** Representative western blots of the active form of ATF6 (left) and the mean expression of ATF6 in amyotrophic lateral sclerosis (ALS) and controls (with 95% CI, right; quantification of protein level by densitometry, adjusted to GAPDH). **(B)** Receiver operating curves (ROCs) to discriminate ALS from healthy controls ATF6. **(C)** The expression of ATF6 is plotted against disease phases relative D50 (rD50). Cluster 1 (red squares) shows increasing expression levels and cluster 2 (green circles) a stable expression across disease phases.

PBMCs from 49 ALS patients and 31 age- and sex-matched healthy controls were studied. Clinical characteristics are provided in Table 1. The cleaved form of ATF6 was significantly increased in ALS relative to healthy controls (*p* = 0.001; Figure 1A). ROC curve to discriminate ALS from healthy controls for ATF6 is displayed in Figure 1B (area under the curve = 0.79, *p* < 0.001, sensitivity 87%, specificity 45%). ATF6 expression levels did not correlate with disease severity (ALSFR-R score), disease duration (onset since first ALS motor symptom), progression rate [(48-current ALSFRS-R)/disease duration in months] and disease aggressiveness (D50). Further, no significant differences in expression levels were observed between patients with either bulbar or limb onset. In Figure 1C, ATF6 expression levels are plotted against rD50-derived disease phases. Two distinct clusters of patients were observed: cluster 1, with progressively increasing expression levels and cluster 2, which demonstrated stable expression over the disease course. Individuals in the two clusters did not significantly differ in terms of ALSFRS-R, D50, rD50, disease duration and subtype. However, patients in cluster 1 (mean age = 49 ± 11 years) were significantly younger than those in cluster 2 (mean age = 61 ± 13 years; *p* = 0.03).

**Table 1:** Clinical characteristics.

		Controls		ALS	
Sex (n, %)	f	14	45.1	21	42.8
	m	17	54.9	28	57.2
Age (mean, SD)		60	12	60	12
ALSFRS-R total (mean, SD)				35	8.1
Onset type (bulbar/limb)				18/31	36.7/63.3%
Disease duration (mean, SD, months)				28	18
Progression rate				0.59	0.49
D50				48	31
rD50				0.34	0.15
ATF6–50 kDa		0.007	0.014	0.063	0.085

*ALSFRS-R: Revised ALS Functional Rating Scale; Disease duration: onset since first ALS motor symptom; Progression rate: (48-current ALSFRS-R)/disease duration in month; D50: time point when ALSFRS-R drops to 24; rD50 = relative D50 describes individual disease course covered in reference to D50; ATF6- 50 kDa: mean expression of ATF6 adjusted to GAPDH*.

## Discussion

We demonstrate here that the UPR pathway *via* ATF6 is activated in PBMCs from ALS patients. This is in line with former studies in animal models and human ALS (Atkin et al., 2008; Prell et al., 2012). Peripheral blood cells dynamically reflect multisystemic changes in the proteostasis pathways in ALS. PBMC of ALS patients showed differential expression of cytoplasmic and ER chaperones (Nardo et al., 2011). A recent study of PBMCs in a ALS cohort, comparable to our cohort, also observed an upregulation of other UPR elements, namely the spliced XBP1 and GRP78 (Vats et al., 2018). However, so far the UPR in PBMCs was not studied in relation to disease progression and disease phases. Although animal models suggest that ER stress is linked to disease progression (Nardo et al., 2013), we observed no association between expression levels of active ATF6 and disease progression. The cluster with increasing ATF6 across disease phases was significantly younger, indicating that aging processes may be related to ER stress in ALS. In general, the ability to respond to environmental stress decreases with age, which is reiterated by the involvement of a perturbed UPR in numerous age-related conditions. For instance, in nematodes and mammals, the ability to activate the UPR in response to stress declines during the aging process (Higuchi-Sanabria et al., 2018). This favors the aggregation of misfolded proteins and ultimately cell death. Conversely, augmentation of the UPR can actually protect neuronal cells during stress and improve longevity (Tanaka et al., 2000; Ko et al., 2002; Saxena et al., 2009; Taylor and Dillin, 2013). Our data suggest that the UPR (*via* ATF6) is diminished as ALS progresses in older patients. We hypothesize that in aged patients with ALS, the UPR is probably less effective than in younger patients. These findings suggest that the influence of age on disease pathology and severity cannot be ignored. In terms of clinical trials a potential neuroprotective effect (e.g., chaperone inducing therapy) and the stratification of patients have to take age-related deterioration of the ER-stress response into account. Longitudinal studies are necessary to prove our hypothesis of diminished UPR in older ALS patients.

## Author Contributions

TP and JG: conception and design of the work. AR, TL and VT: acquisition of data. BS and JG: analysis of data. TP: drafting the work. OW and NG: revising the work critically for important intellectual content.

## Conflict of Interest Statement

The authors declare that the research was conducted in the absence of any commercial or financial relationships that could be construed as a potential conflict of interest.
